# Current Challenges in Monitoring Low Contaminant Levels of Per- and Polyfluoroalkyl Substances in Water Matrices in the Field

**DOI:** 10.3390/toxics12080610

**Published:** 2024-08-20

**Authors:** Hector Medina, Carson Farmer

**Affiliations:** School of Engineering, Liberty University, Lynchburg, VA 24515, USA

**Keywords:** PFAS, low concentrations, sensitivity, sensors, drinking water, compliance

## Abstract

The Environmental Protection Agency (EPA) of the United States recently released the first-ever federal regulation on per- and polyfluoroalkyl substances (PFASs) for drinking water. While this represents an important landmark, it also brings about compliance challenges to the stakeholders in the drinking water industry as well as concerns to the general public. In this work, we address some of the most important challenges associated with measuring low concentrations of PFASs in drinking water in the field in real drinking water matrices. First, we review the “continuous monitoring for compliance” process laid out by the EPA and some of the associated hurdles. The process requires measuring, with some frequency, low concentrations (e.g., below 2 ppt or 2 ng/L) of targeted PFASs, in the presence of many other co-contaminants and in various conditions. Currently, this task can only (and it is expected to) be accomplished using specific protocols that rely on expensive, specialized, and laboratory-scale instrumentation, which adds time and increases cost. To potentially reduce the burden, portable, high-fidelity, low-cost, real-time PFAS sensors are desirable; however, the path to commercialization of some of the most promising technologies is confronted with many challenges, as well, and they are still at infant stages. Here, we provide insights related to those challenges based on results from *ab initio* and machine learning studies. These challenges are mainly due to the large amount and diversity of PFAS molecules and their multifunctional behaviors that depend strongly on the conditions of the media. The impetus of this work is to present relevant and timely insights to researchers and developers to accelerate the development of suitable PFAS monitoring systems. In addition, this work attempts to provide water system stakeholders, technicians, and even regulators guidelines to improve their strategies, which could ultimately translate in better services to the public.

## 1. Introduction

Per- and polyfluoroalkyl substances (PFASs) are a large class of anthropogenic chemicals manufactured since prior to the middle of the 20th century (although carbon tetrafluoride was reportedly synthesized in 1886 [1]) and broadly used in many applications due to their multiple surface active agent (a.k.a. “surfactant”) properties. Also commonly referred to as “forever chemicals”, PFASs are a family of near 15k (this number might be in the few millions, according to some studies [2]) molecules of between ≈50 and ≈3500 Daltons (Da)—with an average of ≈400 Da—and exhibiting a variety of structures such as linear, branched, cyclic, and combinations of those. The presence of relatively strong C-F bonds in these compounds presents a challenge in terms of their decomposition. PFASs have been utilized since the 1940s [3]. These chemicals are engineered to exhibit a diversity of properties, including dual surfactant-like properties such that they can repel—or attract—oil and water, and to exhibit thermal resistance, chemical stability, and other attributes that render them integral to the manufacturing of products such as nonstick cookware, stain-resistant fabrics, and firefighting foams. Unfortunately, accumulating scientific evidence robustly supports the association between prolonged exposure to specific PFAS compounds and an increased risk of cancer and other health disorders [4,5,6,7,8,9]. Furthermore, exposure to these substances during pivotal developmental periods, such as pregnancy or early childhood, is linked to significant adverse health outcomes [10,11,12,13,14], which can be detrimental to the sustainable health of the next generation.

**Figure 1:**
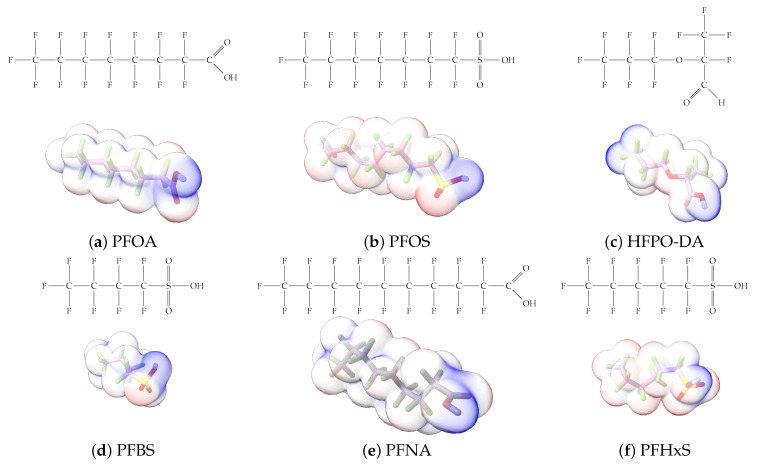
The six molecules that are part of the EPA regulations for drinking water: (**a**) PFOA, (**b**) PFOS, (**c**) HFPO-DA, (**d**) PFBS, (**e**) PFNA, and (**f**) PFHxS molecules. The upper part of each subfigure (**a**–**f**) shows the 2-D chemical structure and the lower part shows the molecular electrostatic potential (MESP) at the 0.001 e/Bohr^3^ surface calculated using tight-binding methods, xTB-GFN2 [16] with Multiwfn [17,18].

Furthermore, studies have found that at least a significant percentage of the US population is potentially exposed to some type of PFASs. Making headlines in the national news, a study conducted by the US Geological Survey (USGS) found that at least 45% of the nation’s tap water is estimated to have one or more types of PFASs [15]. Others have estimated a range of between 54–83% of the US population to be exposed to either perflourooctanoic acid (PFOA) or perfluorooctane sulfonate (or sulfonic) acid (PFOS) (see Figure 1) in their drinking water. However, the types of known PFASs is much larger than those considered in most studies. Recognizing the urgent need to slow down the presence of PFASs in humans and the negative health effects of these molecules, regulatory agencies have issued standards to limit PFAS concentrations. For example, the EPA recently issued national standards to regulate six types in PFASs in drinking water. However, as it is explained in detail in this paper, the implementation of those standards comes with many challenges. For example, an expected large demand for testing services at the concentrations prescribed will most likely put a burden on current laboratories across the nation. Ideally, portable (in situ) sensor technologies would satisfy the foreseeable large demand, but their developments still face many challenges.

In this paper, we address various challenges related to measuring low concentrations—where “low” is with respect to the allowed maximum contaminant levels or trigger levels—of PFASs in water systems in the field. We start by summarizing the recently pioneered regulations issued by the EPA, including the processes prescribed for continuous monitoring (and related remediation). The current methods, techniques, and instrumentation are outlined, as well as some of their imitations in terms of scalability based on a foreseeable high demand. Then, the need for and challenges of the development of portable, highly selective, and sensitive sensor technologies are addressed. As part of those challenges, the paper highlights the issues related to the diversity of chemical structures and properties in PFASs based on the authors’ own computed data via *ab initio* and machine learning techniques, and relying on high-performance computing resources. Next, we outline various sensor technologies that have the potential to overcome the latter, while highlighting some of the technical challenges to their development. We conclude this paper by outlining key points worth further discussions, research, and development.

## 2. EPA’s National Primary Drinking Water Regulations

In April of 2024, the United States (US) Environmental Protection Agency (EPA) announced the first-ever national, legally enforceable drinking water regulation against per- and polyfluoroalkyl substances (PFASs) [19], which is summarized in Table 1. However, concerns for PFASs in drinking water are not a recent matter, and human exposure to PFASs through drinking water has been widely reported in the literature (for a review on the topic, the interested reader is referred to [20]). Initially, only two types of PFASs were the focus of most studies and litigations [21,22]: PFOA and PFOS, see Figure 1. For example, in the US, one of the most investigated cases related to PFAS contamination of drinking water occurred in six water districts in Mid Ohio Valley, West Virginia, near a DuPont chemical plant that used PFOA in the manufacturing of fluoropolymers since the early 1950s [23].

Eventually, at the beginning of 2009, the USEPA issued the first Provisional Health Advisory (PHA) values for PFOA and PFOS at 400 and 200 ppt (parts per trillion), respectively [24]. The equation used to developed those values is as follows:

(1) $$\frac{[\left(NOAEL\right)\;or\;\left(BMDL_{10}\right)]\times BW\times RSC}{UF\times EF\times WI}$$

where NOAEL = no-observed-adverse-effect level; $BMDL_{10}$ = 95% lower bound on the benchmark dose; BW = body weight; RSC = relative source contribution; UF = uncertainty factors; *EF* = extrapolation factor; and *WI* = water intake. (The interested reader can find more details about these parameters in [24]).

These PHA values, developed by the Office of Water (OW), were meant to assess potential risks from exposure to those PFASs through drinking water. At the time, the EPA admitted information on the toxicity of other (than PFOA and PFOS) PFASs was limited, and therefore, no attempt was made to provide corresponding PHA values for any other PFAS contaminants. Those PHA values for PFOA and PFOS were developed to provide information in response to the urgent concerns on the toxicity of PFASs. Later, in 2014, the USEPA drafted two “Health Effects” documents also for PFOA and PFOS. Then, in 2016, the USEPA issued lifetime health advisories (HAs)—which superseded the 2009’s PHA values—for, again, PFOA and PFOS. The HA values were set at 70 ppt for both chemicals [25]. At the time, the EPA acknowledged that both PFOA and PFOS had similar types of adverse effects, and hence recommended that, also, their combined concentrations in water should not exceed 70 ppt. By definition, HAs are not regulations and ought not to be used as legally enforceable standards. In the meantime, drinking water levels multiple times more than the EPA’s lifetime HA values were reported at various locations across the US [26,27]. In June of 2022, the EPA updated the HAs for PFOA and PFOS to more restrictive levels and added two other PFAS types [28]: hexafluoropropylene oxide (HFPO) dimer acid and its ammonium salt (together referred to HFPO-DA, but more commonly known as “GenX chemicals” or simply “GenX”), and perfluorobutane sulfonic acid and its related compound potassium perfluorobutane sulfonate (together referred to as PFBS); see Figure 1. Reportedly, animal toxicity studies had led the EPA to not only restrict the recommended levels of the aforementioned PFAS, but to also include other two types to their more recent list: perflourononanoic acid (PFNA) and perfluorohexane sulfonic acid (PFHxS); see Figure 1. The EPA recently announced the first-ever national primary drinking water regulations (NPDWRs) that establish maximum contaminant levels (MCLs) for a total of six PFAS: PFOA, PFOS, PFHxS, PFNA, HFPO-DA, and PFBS (see Figure 1 and Table 1). Besides MCLs, which are enforceable, the EPA issued recommended—i.e., nonenforceable—MCL goals (MCLGs), which are meant to highlight that current scientific evidence demonstrates that “there is no lower limit level of exposure to these contaminants without risk of health impact, including certain cancers” [19]. In addition, a hazard index (HI)—under both MCL and MLCG—of 1 (unitless) was issued for PFAS mixtures containing at least two or more of of PFHxS PFNA, HFPO-DA, and PFBS. This HI can be calculated using the following formula:

(2) $$HI=\frac{WC_{PFHxS}}{10\;\mathrm{ppt}}+\frac{WC_{PFNA}}{10\;\mathrm{ppt}}+\frac{WC_{HFPO-DA}}{10\;\mathrm{ppt}}+\frac{WC_{PFBS}}{2000\;\mathrm{ppt}}$$

where $WC_{\left(\;\cdot \;\right)}$ refers to the concentration in water of the corresponding PFAS, in units of ppt. In the summary of the NPDWR standards presented in Table 1, note that compliance is determined by running annual averages (RAA) at the sampling point. Also note that significant figures of the values shown are to be strictly observed.

**Figure 2:**
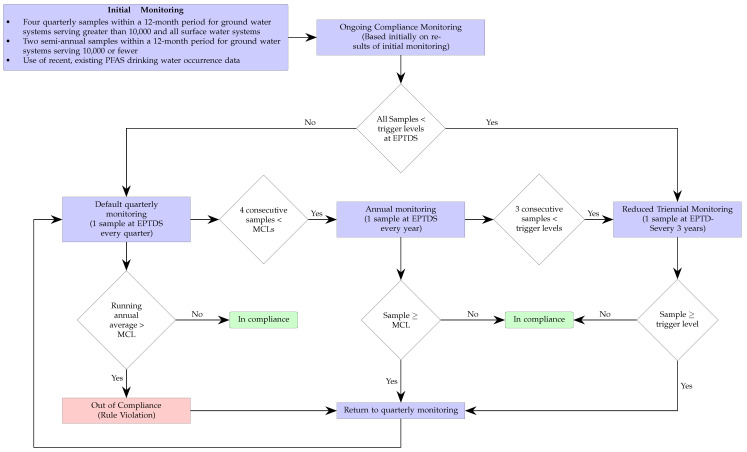
The monitoring frequency for the EPA regulations is highlighted in the flowchart. The sample collection occurs at the entry point to the distribution system (EPTDS). Initial compliance can be based on previously obtained data. The facility would be out of compliance if after quarter monitoring for a year the averages are above the maximum contaminant levels (MCLs). The MCLs are listed in Table 1. The trigger levels are half of the corresponding MCL of the specific PFAS.

The corresponding continuous monitoring for compliance process associated with the NPDWR standards is shown in Figure 2. The initial monitoring could consist of either collecting and testing two or four samples—depending on the system size and type—over a one-year period, or it could simply rely on the use of preexisting suitable data. Based on that initial information, the water system could either default to quarterly or triennial monitoring depending whether the sample levels where, respectively, below or above the so-called trigger levels—which are set at 50% of the MCLs. If the latter, remediation strategies must be implemented to reduced the levels until compliance is reached.

## 3. Current Methods and Instrumentation

The state-of-the-art (SOTA) technologies for measuring PFASs rely on protocols produced by the EPA in collaboration with various sectors (private laboratories, academia, etc.), and they use liquid chromatography (LC) combined with tandem mass spectrometry (MS/MS). The existing analytical methods developed by the EPA are currently Method 533 [29], Method 537 [30], which was nine years later updated to 537.1 [31], and, the most recently released, Method 1633 (January 2024) [32]. All these methods rely on a solid-phase extraction (SPE) step, which allows for the concentration of the sample; this step is followed by analysis with LC-MS/MS, fitted with a so-called “C-18” column. A notable difference between Method 537.1—also referred to as modified 537 (537M)—and 1633, is that while the former was applicable to 70 types of PFASs, the latter has been limited to only 40 types. All these methods can provide detection at levels lower than those stipulated by the new EPA regulations (i.e., ≤4 ppt). For example, limits of detection (LOD) values have been reported in the literature to be ≈0.7 to 3.0 ppt using Method 537M, while ≈1.5 to 15 ppt using Method 533 [29,31]. Furthermore, a total of about 70 PFAS can be detected. However, high run times of the LC-MS/MS analysis step (≈30 min) limit the high-throughput testing rate. Moreover, there is only a handful of companies that provide suitable LC-MS/MS equipment capable of yielding the aforementioned LODs. Such equipment have been used in other life science and forensic applications for several years. However, for PFAS applications, equipment must be free of PFAS-containing polymeric material. This is typically achieved by substituting some parts, such as Teflon-coated solvent lines, with some nonfluorinated, such as polyether ether ketone (PEEK)-based, counterparts. There has been at least one nonconclusive report of ultra-low PFAS concentration leachates from PEEK tubing [33]. Needless to say that suitable LC-MS/MS equipment are laboratory-based (i.e., non portable), somewhat heavy and bulky, and could be sensitive to certain lab-environmental conditions—which can be controlled but often at a high cost.

There are other techniques reported in the literature that have been used to analyze PFASs yet not certified or validated by regulatory agencies [34,35,36,37]. These methods include some variations of LC with MS, ion chromatography (IC), fluorometric detection (FD), and gas chromatrography (GC). Some of these methods (e.g., IC and FD) can provide LODs comparable to LC-MS/MS; however, they could require additional extensive pre-treatment steps.

As mentioned earlier, although a few methods were earlier recommended by the EPA, ultimately, Method 1633, published at the beginning of year 2024, seems to be the consolidation of previous attempts to analyze PFASs in water, although it includes other matrices as described by its title: “Analysis of Per- and Polyfluoroalkyl Substances (PFAS) in Aqueous, Solid, Biosolids, and Tissue Samples by LC-MS/MS”. It was developed by the EPA’s OW in collaboration with the Department of Defense (DoD) and with support from various entities (at least: the Institute for Defense Analysis, in Alexandria, USA, SGS-AXYS Analytical, General Dynamics Information Technology, and Science and Engineering for the Environment, LLC). As in most standard methods, there are many details that technicians should get acquainted with before using it. In the sequel, we provide a brief summary of some key aspects of Method 1633 as it relates to water, and some related challenges.

For aqueous matrices, samples are to be collected using high-density polyethylene (HDPE) containers with either HDPE or polypropylene caps. Samples can be collected as “grab samples” from sources that flow freely, such as effluents. In the case of still waters, one must be mindful of PFAS-enrichment on the surface due to their surfactant nature—they tend to create monolayers of molecules on the water surface up to the critical miscelle concentration (CMC) [38,39,40,41].

Once at the lab facility, the samples could be stored under certain environmental conditions, e.g., temperature (T), light exposure, etc., and for certain limited times: 28 days from collection when stored at T ≤ 6° or 90 days, when stored at T < −20° and in the dark. However, it is recommended that the samples be analyzed as soon as possible. For the actual analysis, samples are spiked with isotopically labeled compound standards and extracted using SPE cartridges, followed by a cleanup using carbon before analysis. The analytes are measured as either in their anions or neutral forms. The actual quantification of the concentration of any target analytite is carried out in reference to an isotopically labeled PFAS standard, followed by a conversion from raw peak areas (in sample chromatograms) to final concentrations. Because of the use of isotope dilution and other related processes, errors in the analyte’s final concentrations may be induced. In the method, there are procedures in place to correct for any losses that may occur during sample extraction, extract cleanup, and concentration. However, if the so-called “recovery” standard percentage does not reach certain specified levels, then the method recommends to correct the problem, re-prepare, extract, clean up the sample batch, and repeat the test. Other key aspects related to this method include the following:

- The method is intended for the targeted analysis of 40 PFASs in various matrices, including aqueous samples.
- It relies on ultra-high-performance (UHP) LC and tandem MS. This combined instrumentation must be operated by either analysts experienced with such equipment or operators under the close supervision of such qualified persons.
- Since the method both calibrates and analyzes PFASs using isotopically labeled standards, linear and branched PFAS may be analyzed as a mixture instead of individually.
- The method offers the flexibility of its modification as long as it leads to an improvement in performance. Examples include improvement in the sensitivity, accuracy, or precision of the results or the reduction of interference.

For compliance in the US, public water systems (PWSs) must rely on Method 1633 or an improved version of it for the monitoring and reporting of PFASs in water. The combined protocol with LC-MS/MS-based analytical procedures has been proven to provide the LODs established in the MLCs of Table 1. However, their implementation is restricted to centralized labs. The idea of deploying certified, LC-MS/MS fully equipped labs at each PWS seems unfeasible and is limited by the expensive cost of the instruments and the requirements of laboratories with trained personnel to run them. Furthermore, outsourcing regular monitoring—although the current *solus via*—could soon become a burden due to the high costs of USD 200–600 for each sample, which also prevents broader sampling and testing of common PFASs. This state-of-affairs could encourage (especially the smaller) PWSs to avoid carrying out extra tests, which otherwise would be beneficial for higher-confidence reporting and for the good of the general public. Hence, more straightforward, quicker, less expensive, and ideally field-based approaches are needed to accurately and more rapidly assess the danger of PFAS exposure to people. This need for PFAS monitoring may be accomplished by portable transducers or sensors, which react to an analyte’s presence and convert that information into a signal that can be processed (e.g., amplified and filtered) and used in real time. Even though PFASs are present in various matrices and their detection is important regardless of the matrix, more frequent detection of PFASs in aqueous matrices is a useful place to start when assessing both the exposure to human health as well as the distribution and transport of the various types of PFASs. However, as it will be presented in the sequel, the development of portable, low-cost, robust detecting systems that can be easily deployed in the field and withstand realistic interference is still very challenging.

## 4. Diverse Chemical Structures and Properties

A critical aspect of the problem of detecting low concentrations of selected PFASs stems from the fact that there are many more of those contaminant molecules (≈15,000, or perhaps millions [2]) than the few currently regulated by the EPA. The design of detection systems with high selectivity must account for this. In addition, the diversity of compositions, structures, and properties render the problem of designing robust sensors a very difficult task. For example, various PFASs can exhibit a variety of molecular electrostatic potentials (MESPs) as shown in Figure 1 for the six PFASs regulated in the NPDWRs, and where the MESPs are obtained at the Bohr’s surface via tight-binding theory (xTB-GFN2) [16] with Multiwfn [17,18]. In addition, molecular moieties and structures in PFASs can widely vary, which leads to diversified ways of classifying them. For example, the molecular structures of PFASs can be classified as linear, branched, and cyclic, as shown in Table 2. For classification purposes, linear molecules are considered to be any molecule with a continuous $CF_{2}$ chain. Branched molecules contain a $CF_{2}$ or $CF_{3}$ group in separated portions of the molecule. Cyclic elements contain at least one ring with at least one fluorine-containing subgroup attached to the ring. These structures can have important effects on the way PFASs interact in humans, biota, and the environment in general [42,43,44,45,46,47]. For example, aromatic PFASs (such as flurophenolic compounds), a type of cyclic structures, have been biodegraded using various peroxidases routes as catalyzers [48,49,50]. However, this mechanism has not appeared to be very successful for some other non-aromatic types of PFAS, and biodegradation mechanisms for some promising microorganisms still need to be elucidated [51].

Various functional groups also seem to be of common occurrence in PFASs due to their surfactant-like, intended behavior. Shown in Table 3 are a selection of functional groups—such as carboxylic acid and sulfonic acid—and examples of the corresponding complete PFAS molecules. In fact, it is suspected that not only could these functional or head groups play a critical role in binding with living organisms but they could also be the “weak points” to promote chain-reaction-like decompositions of PFASs, specially in bioremediation pathways (see preprint reference [52] of the authors’ work in progress on the topic of bioremediation). Included in Table 3 are also the number of PFAS molecules from the EPA’s database list containing such functional groups as well as the corresponding IUPAC (International Union of Pure and Applied Chemistry) names. Various approaches to sense and adsorbed PFASs have been based on their functional groups [53], a technique that has also been used for degradation mechanisms [54].

The ionic nature of PFASs confers them the ability to interact as well with aqueous systems as with other media such as oil or oil-like substances. Some PFASs, therefore, exhibit strong hydrophobic and hydrophilic behaviors, and these can vary based on the conditions of the environment. These behaviors can contribute greatly to their toxicity, mobility in the environment, and bioaccumulative nature. In addition, these characteristics can help guide protocols for water sample collection and other steps during the monitoring and remediation processes. For example, water surface PFAS enrichment can be explained using the principles of critical micelle concentration (CMC), which is a parameter of surfactants such as many PFASs; but the CMC varies with water temperature, pressure, pH, etc. From an ionic perspective, PFASs can be classified as either noninoic or ionic, and the latter can, in turn, be sub-classified as anionic, cationic, and zwitterionic; see Table 4. The ionic type can influence various aspects of the design and development of detection systems as well as remediation materials. For example, the design of sensors and/or adsorbing materials aimed at interacting with zwitterionic PFASs must strictly consider the range of operating pH values. Finally, the ionic groups play a role in the interaction of PFASs with biological systems, potentially leading to bioaccumulation and adverse health effects.

Another important classification of PFASs that is critical to the development of suitable sensor systems is related to their molecular size, specifically the number of carbons contained in the chain. Typically, PFASs have been classified as ultrashort (1 or 2 carbon atoms), short (3 to 6 C), and long (7 or more C). Table 5 shows groups of PFASs organized based on the size of their chain length. There are studies on the effects on the ability to detect or adsorb PFASs depending on their chain length [55,56]. For example, it has been reported that while conventional adsorption-based removal mechanisms—for example, using activated carbon—of PFASs from water can be effective for long-chain molecules, they are not as effective for their short-chain counterparts [57]. Furthermore, there have been reports of elevated levels of ultrashort- and short-chain perfluoroalkyl acids (PFAAs) in homes in the US [58], which should be an important concern, but perhaps also an opportunity for home-water-filtration solution developers.

**Figure 3:**
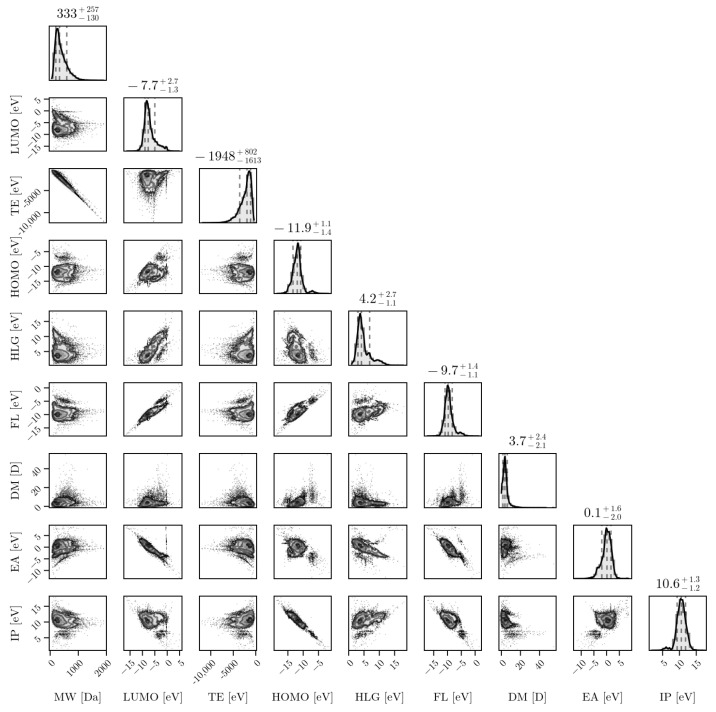
From the EPA’s list (PFASStructV5), the molecular properties are shown through their relationships with one another. The plot at the top of each column represents a histogram of a single property. The properties include MW (Molecular Weight), Lowest Unoccupied Molecular Orbital (LUMO), TE (Total Energy), Highest Occupied Molecular Orbital (HOMO), HOMO LUMO Gap (HLG), Fermi Level (FL), Dipole Magnitude (DM), Electron Affinity (EA), and Ionization Potential (IP). Units are either in electron-volts (eV) for all energy-based properties, Daltons (Da) for the MW, or debyes (D) for the DM.

The plurality of compositions and structures discussed above leads to diverse distributions of properties, which render the problem of designing robust and generalizable PFAS detection systems even more difficult. Figure 3 shows a corner plot for a set of distributions of PFAS properties obtained via *ab initio* computational methods for almost 15,000 PFAS molecules. The properties shown in Figure 3 are as follows: molecular weight (MW), in Daltons (Da); dipole moment (DP), in debye (D); and the total energy (TE), the HOMO-LUMO gap (HLG), the Fermi level (FL), the electron affinity (EA), and the ionization potential (IP), all in electron-volts (eV). The structures were generated based on the InChI strings provided, and salt ions were removed prior to structural optimization. All of the structures were optimized at the GFN2-xTB level of theory [16]. These properties are important in understanding the behaviors of PFASs, particularly when comparing against properties of potential candidate molecules for sensors and remediation materials. In order to develop selective technologies, tools should be produced to properly cluster and analyze groups of PFASs based on various structures and properties (which is part of a work in progress by the authors). Such tools could provide a means to accelerate discovery via HPC and a combination of *ab initio* algorithms with suitable machine learning architectures such as graph neural network-based approaches (work in progress).

## 5. PFAS Sensors: Field-Based Portable, Low-Cost, Monitoring Technology

Without a doubt, having low-cost, reliable, portable testing apparatuses (in the sequel referred to as PFAS sensors) can be very beneficial. Granted, central laboratory facilities will be needed for a long while to carry out robust testing, especially given the fact that EPA methods are based on LC-MS/MS equipment only. In addition, these laboratory facilities can provide validation for the development of new sensors and new procedures, at least. However, the development of PFAS sensors with high fidelity and sufficient LOD—single-digit ppt—is not an easy task. For example, the ability of a PFAS sensor to distinguish between the target molecule and other competing contaminants, or even other PFASs, is very important. Such *interference* of other molecules is an important aspect that must be considered in the design of good PFAS sensors. Hence, a good PFAS sensor must exhibit high *selectivity*, which is its ability to distinguish between the targeted analyte and interfering molecules—including other nontargeted PFAS molecules. Finally, a reliable PFAS sensor must exhibit sufficient *sensitivity*, which means that at any given transduction, its signal-to-noise ratio (SNR) is sufficiently large for a targeted LOD.

### 5.1. Interference, Selectivity, and Sensitivity

**Interference:** Interference could come from various other water polluting molecules, including other PFASs not being targeted by the sensor [59,60,61,62,63,64,65,66,67]. The development of PFAS sensors and measurement protocols must include validation in a realistic distribution of lab-spiked matrices. Among the competing contaminants are chloride, humic acids (a mixture of organic compounds originating from the degradation of living matter), flouride, various dyes, etc. In general, contaminants that could interference with PFASs could fall under one or more of the following categories:

- Biological: These include bacteria (such as *E. coli* and *Salmonella*), viruses (such as norovirus and hepatitis), and parasites (such as *Giardia* and *Cryptosporidium*). These pathogens can cause diseases ranging from mild gastrointestinal distress to more severe conditions [59,60].
- Chemical:
  – Organic Chemicals [61,62]: These can include pesticides, herbicides, and industrial chemicals such as benzene or polychlorinated biphenyls (PCBs). Many of these chemicals enter water sources through agricultural runoff, industrial discharge, or leakage from waste disposal sites. Other chemical contaminants can come from medication, cosmetics, and personal care products.
  – Inorganic Chemicals [63,64]: Common examples include heavy metals like lead, mercury, arsenic, and cadmium, as well as fluoride, nitrates, and nitrites. These can come from natural mineral deposits, industrial processes, and agricultural practices.
  – Compounds: Chemicals used in the water disinfection process, like chlorine, can react with natural organic materials in water to form byproducts such as trihalomethanes (THMs) and haloacetic acids (HAAs).
- Radiological [65,66,68]: Naturally occurring or man-made radioactive substances like cesium, radium, uranium, plutonium, or radon can contaminate water sources.
- Physical [59,67]: These could include other suspended solids or total dissolved solids that are only mechanically mixed with the water matrix.

**Selectivity**: Besides the various interfering molecules, a good PFAS sensor must overcome the challenge of selecting targeted analytes within changing environmental conditions such as variations in temperature, pH, and ionic strength, all of which can affect the transduction behavior of the sensor.

**Sensitivity**: Regardless of how highly selective a PFAS sensor is, its LOD might be limited by its sensitivity, which refers to the steady-state gain between the output variable and the actual measured property by the probe. The higher this ratio is the more sensitive the sensor would be to small changes in quantities of the analyte (i.e., the lower the concentrations it can measure).

### 5.2. Other Considerations

**Stability and Durability**: Sensors must maintain their performance over time and under cycling environmental loading conditions. The stability and durability of sensor materials are crucial for reliable long-term operation.

**Cost and manufacturing tunability**: Developing cost-effective PFAS sensors that can be produced at scale is essential, especially since water monitoring applications require widespread worldwide deployment. Also, during the fabrication processes, it is desirable to be able to tune sensor properties such as sensitivity and selectivity.

**Ease of operation, maintenance, calibration**: Easy-to-operate PFAS sensors ensure that a wide range of users, including those with limited technical expertise, can use them. Also, simple operations reduce the costs of training or of hiring specialized personnel. In addition, sensors that are simple to operate could engage the general public in their adoption and domestic use. In the case of nondisposable sensors, maintenance, including frequent calibration, must be considered. The cost of calibration (or maintenance) procedures and personnel could be high in cases where highly specialized equipment is required, for example. Cleaning and maintenance can be required more often (than regularly prescribed) if the sensor experiences fouling more frequently due to the unusual presence of co-contaminants or organic matter, minerals, etc.

### 5.3. PFAS Sensor Technologies

There are several PFAS sensor technologies either developed or under development. Table 6 summarizes a list of various technologies included in this paper. For a more exhaustive list, the interested reader can refer to reviews and other papers focused on this topic [34,69,70,71]. In the sequel, we provide a brief explanation of the fundamental technology followed by a short survey on the development, for each technology.

#### 5.3.1. Molecular Imprinted Polymers

Molecular imprinted polymers (MIP) are a type of synthetic materials designed to mimic natural recognition entities—analogous to antibodies and biological receptors. They are synthesized with specific cavities tailored for a target molecule. The cavities are created during the polymerization process in the presence of the target molecule acting as a template [101]. Typically, they are synthesized following some variation of the following steps:

- Template molecule introduction [102]: The target molecule, which the polymer will be imprinted to recognize, is mixed with monomers.
- Polymerization: This mixture is then polymerized in the presence of cross-linkers. The template molecule influences the positioning and orientation of the monomers as they form the polymer matrix [103].
- Template removal: After polymerization, the template molecule is removed, leaving behind cavities that are complementary in shape, size, and functional groups to the template [104].

**MIP technologies for PFAS sensors**

MIP sensors [72,73,74,75,76,77,78,79,80,81,82,83,84] offer several advantages in comparison to other technologies. For example, besides being stable for a practical range of environmental conditions such as pH, pressure, and temperature, MIP PFAS sensors also exhibit good sensitivity and selectivity [72,73,74]. The selectivity is an intrinsic characteristic of MIP sensors due to the utilization of a combined template/analyte in their fabrication [105]. In addition, MIP PFAS sensors could rely on a variety of transduction mechanisms such as electrochemical [78,80,106], photoelectrochemical [83], potentiometric [76], electrochemiluminescence [77], and others. Furthermore, other customizations can be obtained by further functionalization of the polymer matrix. For example, by functionalizing the base polymer with electroactive monomers, the MIP can be made to detect the (nonelectroactive) PFASs via electrochemical/potentiometric transduction [76]. Another type of customization can be obtained by doping the polymeric template with some type of quantum dots (QDs) such as fluorescence QDs. QDs are semiconductor nanoparticles that exhibit unique optical and electronic properties such as a sharp emission profile, high photoluminescence and photostability efficiency, and size-dependent emission wavelengths, which make them appropriate for analytical sensors applications [107,108]. Due to all the aforementioned benefits, among all PFAS sensors, MIPs seem to provide the lowest LODs. For example, using an ultrathin $C_{3}N_{4}$ nanosheet as substrate surface and Polypyrrole as the MIP template, PFOA was reportedly detected at an LOD of about 10 ppt within a concentration range of 20 to 40,000 ppt (note: for the sake of easy comparison against the EPA’s MCLs, we consistently use parts per trillion (ppt) in this work) [77]. Furthermore, a chitosan hydrogel-based MIP sensor doped with carbon quantum dots (CQDs) [107,109,110] and made for PFOS detection was reportedly able to detect LODs of 0.0004 ppt, which is remarkably much lower than any current LC-MS/MS systems [85]. However, since the hydrogel bead is likely destroyed during elution of PFOS using NaOH, the sensor is not reusable [85].

#### 5.3.2. Nanoparticle-Based Sensors

Nanoparticles (NPs) find various definitions in the literature, but mostly they refer to materials that exhibit at least one dimension with size between 1 and 100 nm. Under this broad definition, a carbon nanotube of micrometer length can be considered a nanoparticle. A more strict definition limits the NP to have all dimensions bounded to the 1–100 nm range, or the so-called 0-dimension materials—which is the definition used in this paper. Under this classification, two subclasses are recognized in the literature depending on size: from 1 to 10 nm, QDs, and from 10 to 100 nm, simply NPs. Hence, NP-based sensors could be related to 10–100 nm-sized materials (hereinafter referred to as large NP-based sensors) or 1–10 nm-sized materials (hereinafter referred to as QD-based sensors). The main idea of these sensors is that they exploit the benefits of either bare or functionalized NPs (i.e., NPs with ligands attached to them). NPs display unique properties not found in their polyparticle, bulk-sized, counterpart materials. NPs have been extensively investigated in electronics, photonics, polymer nanocomposites (PNC) [111,112], biomedical sensing focused primarily on disease marker detection [113], environmental detection [86,89,90,91,92,114,115], and biotechnology in general [116]. Other advantages offered by NP-based sensors include the following: (i) their strong physical affinity to confine or trap electrons/holes, thus reshaping the density of states distribution near the bands, a phenomenon that the authors have recently observed in ongoing QD-based PNC research; (ii) their large surface-to-volume ratios can help tailor their sensitivity; (iii) they have chemically tailorable physical properties, which are directly related to their size, composition, shape, and functionalization [117].

**Large NP-based sensors**

Perhaps the two most reported Large NP (LNP) PFAS sensors are based on either Au or $Fe_{3}O_{4}$ NPs. The exceptional chemical robustness, size-contingent optical characteristics, and electrochemical behavior of gold nanoparticles (AuNPs) have positioned them as a paradigmatic nanoparticle in various research domains, including sensing [90,118,119]. For fast response and easy-to-fabricate PFAS sensors, AuNPs offer various advantages such as biocompatability to aqueous media, ease of surface functionalization, and fast electron transport, which make them appealing as candidates for colorimetric or electrochemical transduction [120]. AuNPs have been functionalized with thio-terminated polysterene or single layers of alkanethiolates terminated with PEG-thiol (polyethylene glycol thiol) as well as perfluorinated thiol [89,90]. Perhaps, the best results so far have been those reported by [90] with an LOD of around 10,000 ppt, which is several orders of magnitude higher than the MCLs issued by the EPA. The aforementioned detector was tailored for large C-F chain PFASs.

On the other hand, magnetite $Fe_{3}O_{4}$ NPs owe their interest in the environmental sensing field to their magnetismm, peroxidase-like (or photocatalytic) properties, stability, and biocompatability [121,122,123]. Furthermore, nanocomposites that combine magnetite with molybdenum disulfide ($MoS_{2}$) were developed, characterized, and shown to exhibit augmented catalytic activity [124], which is the property exploited in developing PFAS colorimetric sensors with a relatively simple operation and low cost. The augmented surface area of other so-called hierarchical surface area structures (HESAS) with high catalytic activity should also be explored [125,126,127]. For example, using an absorption mechanism on a microplate reader, PFOS was detected at LODs near 4300 ppt using $Fe_{3}O_{4}$ NPs covalently bonded to $MoS_{2}$ [92].

**QD-based sensors**

QD-based PFAS sensors have also been explored. (Note that these differ substantially from the MIP-based QD sensor mentioned earlier.) Stabilized cadmium sulfide QDs (CSQDs) have been used for the detection of PFOA [86]. The stabilization—accomplished using 3-mercaptopropionic acid—consisted in making the CDQDs hydrophilic, thus allowing for their use in aqueous matrices. The LOD reported for this sensor was 124,200 ppt, which is again much higher than the EPA’s 4 ppt for PFOA. Better LODs have been obtained using CQD-based sensors for detecting PFOS. For example, a CQD-based sensor was fabricated by hydrothermal synthesis with phosphoric acid and o-phenyleneamine that resulted in a device with three measurement signals: pH-sensitive fluorescence emission, absorption, and resonance light scattering, yielding LODs of 9130, 37,900, and 60,200 ppt, respectively. CQDs have also been doped with other elements such as Ni, Br, S, and P to manipulate their emission characteristics. For example, in [87], CQDs were doped with Ni for the ratiometric detection of PFOS, with ethidium bromide added to the mixture of PFOS and CQDs. Using a fluorescence spectrometer, an LOD of 13,900 ppt was obtained, which seems to be one of the lowest in the literature. There has been reports, however, of at least one lower LOD achived using a non-doped CQD complexated with berberine chloride hydrate (BH) [88]. The CDQ-BH complexation was tested in PFOS-spiked water samples and was able to achieve an LOD of 10,800 ppt.

#### 5.3.3. Biosensors

Biosensors have been reported in the literature for the removal of various water pollutants [93,128,129,130]. These sensors can also rely on previously discussed technologies such as NPs/QDs for their functionality. Perhaps, the general basic concept of a biosensor was first introduced in the early 1960s with the so-called “enzyme electrode” by Clark [131,132]. In essence, biosensors rely on biological transductions for sensing the target analyte and producing a physico-chemical response that can be converted into useful information. Some have agreed that there are two general technical strategies used in the design of biosensors, namely, label-based and label-free detection [131,133]. Basically, in label-based detection, the design is based on the specific properties of label compounds to target detection. Biosensors designed using the label-based paradigm tend to offer high sensitivity but tend to be more complex, often requiring combinations of specific sensing elements manufactured with immobilized target proteins. In contrast, the label-free detection paradigm allows for designing biosensors that target molecules that are difficult to tag or are not labeled; also, in some cases, they can be enabled to detect several target molecules simultaneously [131].

There are various types of bisonsensors; yet, the literature does not seem to classify them more clearly than the two paradigms described above. However, in the literature we find *aptasensors*, which are a type of biosensor that use aptamers as the recognition element to detect target molecules. Aptamers are short, single-stranded DNA or RNA molecules that can fold into unique three-dimensional structures, allowing them to bind selectively and with high affinity to specific targets, such as proteins, small molecules, and even cells. Also, there are *Immunosensors*, which are typically designed as an inspiration from how the analyte acts in the human body. For example, some immnusensors use antibody proteins and their mechanism of binding to some antigens (i.e., the target analyte) or vice versa if the target analyte happens to be an antibody. *Enzymatic sensors* utilize enzymes as the biological recognition element to detect specific analytes via exploting the catalytic activity of enzymes to produce a measurable signal. The higher the signal, the higher the concentration of the target substance; at least, that is the intention. Even if the specific enzyme and metabolism route is not completely known, biosensors can be made using full cells or microorganisms.

**Figure 4:**
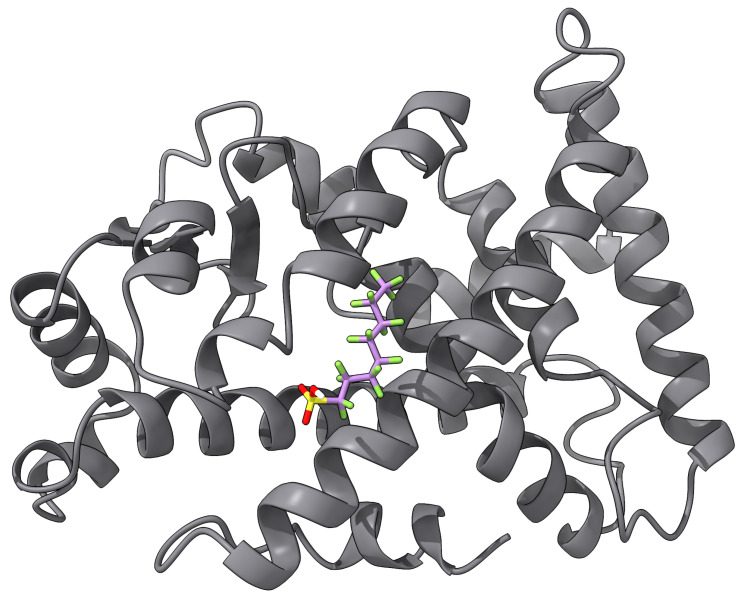
An example of ligand docking of PFOS into PPARα.

Several biosensors have been demonstrated to detect some type of PFAS to some levels. An impedance-based biosensor using human serum albumin (HSA) covalently attached to pyrrole-2-carboxylic acid (Py-2-COOH) with a graphite screen-printed electrode was reported in [94] as a proof of concept. They reported LODs of 207,000 ppt for PFOA, but it was not tested using real samples. An enzymatic biosensor for PFOS was developed using multiwalled carbon nanohorn-modified glassy carbon electrodes for both positive and negative terminals, with glutamic dehydrogenase and bilirubin oxidase as the respective catalysts [95]. They reported an LOD of 800 ppt for PFOS. Furthermore, they claimed that their biosensor is selective for PFOS as other PFASs such as PFOA, PFBS salt, PFOSA, and PFNA did not exhibit any interference. The aforementioned designs and others in the literature seem to exhibit LODs that are far from the MCLs required by the EPA’s NPDWR. Better LODs have been accomplished by biosensors that make use of PFAS binding to some peroxisome proliferator-activated receptor (PPAR), such as PPARα [96,97]. Figure 4 shows an illustration of the docking of PFOS onto PPARα (PDB ID: 4BCR [134]). The docking was carried out with AutoDock Vina [135] with the pocket centered at x=10.97Å, y=4.64Å, z=7.56Å, with sides of length 7.5 Å. The estimated binding energy was −3.775 kcal/mol. For example, gold NPs were modified with PPARα-responsive elements, and Ag was added to enhance the signal-to-noise ratio of the system. Once attached to the microplate, this immunosensor was used to detect PFOS at an LOD of 5 ppt [96]. Furthermore, another immunosensor was designed using QDs modified with a tetrametric bacterial protein isolated from streptomyces avidinii [97]. The modified QDs, serving as a fluorescent marker, bind to the analyte-activated—in this case, PFOS—PPARα complex. The higher the PFOS concentration, the higher the fluorescent intensity of the QDs. This biosensor yielded a remarkable 2.5-ppt LOD. However, none of the PPARα-based biosensors are meant to be deployed in the field, since they require many reagent addition, washing, and incubation steps that can last for hours [69].

#### 5.3.4. Framework-Based Sensors

Metal- and covalent–organic frameworks are highly-organized, porous, hybrid materials with large surface areas and tunable properties. While in a metal–organic framework (MOF) the nodes are metal ions or clusters and the linkers are organic ligands, in a covalent–organic framework (COF), the nodes are light atoms and the linkers are simply covalent bonds. Consequently, both MOFs and COFs can also be viewed as generalized classes of crystalline porous polymers with highly tunable structures, and, hence, properties [136,137,138,139].

A key advantage of MOF/COF-based PFAS sensors has to do with the large amount of “binding” sites that these materials offer due to their high porosity and surface area. This site-enhancement can be translated into the design of sensors with acceptably lower LODs [98,100]. For example, an MOF-based sensor designed to detect PFOS using chromium ions in the center was reported with an LOD of 0.5 ppt [98]. To enhance the signal-to-noise ratio, an electrochemical transducer consisting of an interdigitated microelectrode array was implemented. Also, a COF-based sensor utilizing so-called functionalized lanthanide upconversion NPs was developed to detect PFOSs. Using a flourescent spectrometer, it was observed that the fluorescence of functionalized COF was highly sensitive to the presence of PFOS—and not as much to the presence of PFHxS, PFDA, PFNA, PFOA, or PFHxA. The sensor was tested to sense ultra-trace PFOSs in water and food packing materials, and an impressively low LOD of about 0.075 ppt was achieved [100]. Unfortunately, these lab-based proof-of-concepts have not been validated in the field using real water matrices in order to test for selectivity and interference resistance. Furthermore, when some level of discrimination has been achieved such as in [99], it has come at the cost of very high LODs (e.g., 40,000 ppt in [99]). It might be worth highlighting that regardless of the many advances in diverse technologies, the technology readiness level (TRL) of these and other concepts is still very low.

## 6. Discussion and Future Outlook

The off-site, laboratory-based protocols (e.g., Method 1633) will continue to be, at least for the near future, the *solus via* for PWS to test their water samples for monitoring and potential remediation. For the ongoing compliance monitoring of all surface water systems, it is required that if the monitoring samples (at any monitoring location) exceed the so-called “rule trigger level (RTL)” (50% of the MCLs), frequent (e.g., quarterly) monitoring must be maintained. Depending on the location (with respect to the PWS or remediation facility) and the order backlog experienced by testing laboratories, this could take between 1 or 2 weeks and a few months from sample collection to final results and analysis. Sample collection itself could be very complicated when trying to avoid contaminating the samples with as little as 2 ppt (which is the the RTL) for PFOA or PFOS, 5 ppt of GenX, or 5 ppt of PFHxS, since PFASs can be found in cosmetic products or even in lints from clothing [140,141]. Therefore, care during sample collection and redundant testing are recommended, which could unfortunately further increase both the lead time for the results and the cost of the testing process. Currently, certified testing laboratories can charge approximately between USD 200 and 300 US [142], in current dollars. This cost can, in practice, be much higher (reaching close to USD 600 in some cases) when considering costs from consulting service providers as inter mediators, high-demand effects, and inflation. Ultimately, the extra costs related to continuous monitoring for compliance—and later remediation—will be borne by the end users.

Therefore, it is critical to increase the TRLs of the various laboratory-based proof-of-concept PFAS sensor designs. At the proper TRL, PFAS sensors could at least allow for the following:

- Routine monitoring could be carried out, which would allow for more frequent testing and reporting. Fast detection methods could be implemented, which would help identify more quickly critical areas of PFAS contamination that require immediate remediation attention.
- The general public could potentially bring testing to their homes, which could help expedite implementations of home-based filtration systems. Furthermore, this could help mitigate psychological effects caused by the PFAS problem.

In advancing PFAS sensor technologies, emphasis should be placed on those that promise sufficient sensitivity, selectivity, interference resistance, robustness, and low costs. It appears as though MIP-based sensors, especially those doped with QDs, offer the potential of providing the lowest LODs. In terms of transduction mechanisms, the fluorescent and electrochemical routes seem to have the attention of researchers and developers, and they offer good detection ranges. The need for reusability and robustness to harsh environments could advance the development of enzymatic biosensors, since enzymes and proteins could be tolerant to deactivation by interfering contaminants. The diversity of molecules present in water matrices adds complexity to the development of efficient and robust sensing technologies, though. So, how far are we from having a variety of commercially available PFAS sensors? Unfortunately, only a few patents have been granted or are being considered, despite the large number of academic research works on PFAS sensors. Perhaps, this emphasizes the significant “valley of death” gap between academic research and industrial innovation, and the need to promote more intentional efforts to close that gap.

Without a doubt, the regulation of six PFASs under the recent NPDWR is a milestone step towards ultimate full environmental remediation. It is likely, however, that the number of regulated PFASs will grow in the coming years. But it is also expected that the number of molecules recognized as PFASs will also increase by orders of magnitude [2]. Research on developing tools to elucidate nontargeted PFAS molecules from MS/MS outputs is increasing [143,144]. For example, in silico fragmentation libraries that are based on known and/or computed fragmentation patterns—i.e., collision cross sections—can help in identifying new PFAS compounds where no analytical standards exist [145], but the state-of-the-art still yields too much uncertainty. In any case, the large number of existing PFASs as well as the diversity of their structures and compositions make the consolidation of their properties and interactions with other materials a complex task. This is a very important problem to solve not only for the sake of developing optimal detection technologies, but more importantly, for the purpose of devising remediation solutions that can take care of many PFAS contaminants simultaneously. More studies on the health impacts of larger sets of PFAS will also be needed as evidence to support the urgency of this problem. Ultimately, research, development, innovation, and regulation activities must be aimed at improving the well-being of our communities and the environment. Beyond the challenges of detection and continuous monitoring (which are the main focus of the current paper), it is expected that any monitoring location that exceeds the MCLs must undergo remediation. According to the EPA [19], the implementation of their NPDWR standards by each and every PWS can be outlined as follows:

- The PWS must conduct initial and ongoing compliance monitoring for the regulated PFASs.
- The PWS must implement solutions to reduce regulated PFASs in their drinking water if levels are above the MCLs.
- The PWS must inform the public of the levels of regulated PFASs measured in their drinking water and if an MCL is exceeded.

The EPA has allocated 5 years—from the time the standards were issued—for PWSs to remediate drinking water in case the MCLs are exceeded. The typical methods used to remove PFASs from water include granular activated carbon (GAC), ion exchange resins (IESs), reverse osmosis (RO), etc. From the PWS’s perspective, once the PFAS has been removed from the water system and the MCLs have been met, the PWS’s responsibility is complete. Ironically, there are currently no standards for the PFAS-contaminated material to be disposed of, which means that the PFAS molecules removed from the water could end up in a landfill and leached through back to the drinking water system. However, it seems that this will change for the better soon based on some actions taken by the EPA [146]. In any case, the complete destruction or decomposition of many (if not all) PFAS contaminants should be the ultimate goal of any effective remediation solution, a task that does not seem yet attainable by any of the remediation technologies being openly proposed currently.

## 7. Summary and Conclusions

Although much evidence of PFASs’ negative effects on the health of humans and biota has been reported for decades, regulations have been lagging. Motivated by the recent pioneering NPDWRs by the US EPA to limit the amount of six types of PFASs in drinking water systems, in this paper, we address the most important challenges associated with monitoring low contaminant levels in water matrices in the field. The current Method 1633 accompanied by the associated LC-MS/MS techniques dictate the combined on/off-site processes and the high costs associated with continuous monitoring for compliance. Key to these processes is proper sample collection, which must be executed with extreme care as samples can be susceptible to contamination even directly from the field technician.

The development of robust, selective, highly sensitive sensors capable of low LODs is very desirable. Many design ideas and proofs of concepts have been reported in the literature, some of which have exhibited great potential for suitable sensor systems. However, their maturity level, or TRL, is still relatively low, and the path and time to commercialization are still uncertain on average. There are many challenges that still need to be overcome, many of which stem from the diversity and large number of PFAS molecules. In addition, the conditions of the water sources, including presence of co-contaminants, temperature, turbiditty, pH level, etc., must be accounted for in the design of high-fidelity sensors. Based on the current state of the development, it seems as though MIP-based sensors and biosensors could offer the highest potential. It is recommended that more analytical tools based on computational chemistry (e.g., ab initio DFT, molecular mechanics, etc.) and accelerated via machine learning should be developed accompanied (and validated) by high-throughput experimental data collection. These tools could help consolidate large amount of properties of PFAS molecules and provide a means to accelerate the discovery of efficient material systems and transduction mechanisms.

## Figures and Tables

**Table 1:** A summary of the USEPA’s first-ever National Primary Drinking Water Standards for PFAS for the maximum contaminant level (MCL) and maximum contaminant level goals (MCLGs), as well as the hazard index.

PFAS	MCL (in ppt or Unitless *)	MCLG (in ppt or Unitless *)
PFOA	4.0	0
PFOS	4.0	0
PFNA	10	10
PFHxS	10	10
HFPO-DA (GenX)	10	10
A mixture of 2 or more:		
PFHxS		
PFNA	1	1
GenX	(hazard index)	(hazard index)
PFBS		

(*) in units of ppt for individual PFAS standards and unitless for a PFAS mixture’s hazard index. Compliance is determined by running annual averages (RAA) at the sampling point. Note that the value “4.0” is meant for two significant figures, while the values “10” and “1” for a single significant figure.

**Table 2:** A selection of the linear, branched, and cyclic PFAS molecules are presented. Chemical Abstract Service (CAS) numbers are provided for each structure, and the corresponding names are included in Appendix A.

Linear	Branched	Cyclic
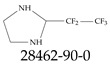	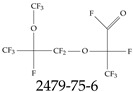	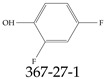
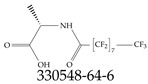	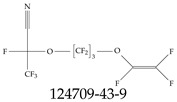	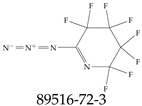
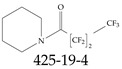	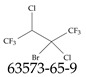	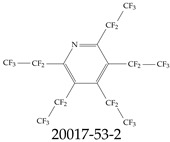
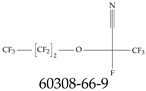	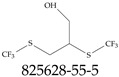	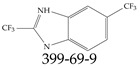
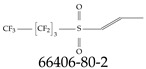	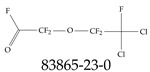	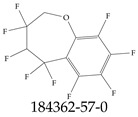

**Table 3:** An example selection of some of the functional groups present for a variety of PFASs. The symbol *R* represents the connection point to the remaining structure. The count refers to the amount of PFAS molecules within the EPA’s list (the so-called PFASStructV5 dataset) that contain such functional group. The International Union of Pure and Applied Chemistry (IUPAC) name is provided for the form where the connection point is replaced with a hydrogen. The last column on the right shows an example of a PFAS molecule containing such a functional group. Chemical Abstract Service (CAS) numbers are provided for each structure, and the corresponding names are included in Appendix A. A note on “NOCAS” identifiers is included in Appendix A, as well.

Functional Group	Count	IUPAC Name	Example PFAS Structure
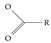	270	formic acid	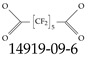
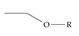	19	methoxyethane	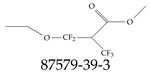
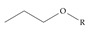	6	propan-1-ol	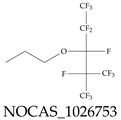
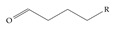	1	Butanal	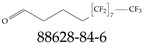
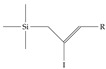	1	(2-iodoprop-2-en-1-yl)trimethylsilane	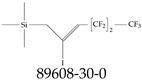

**Table 4:** A selection of the nonionic, anionic, cationic, and zwitterionic PFAS molecules from the EPA’s list. Chemical Abstract Service (CAS) numbers are provided for each structure, and the corresponding names are included in Appendix A. A note on “NOCAS” identifiers is included in Appendix A, as well.

Nonionic	Anionic	Cationic	Zwitterionic
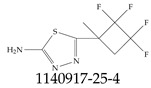	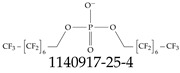	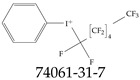	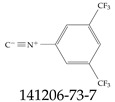
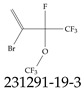	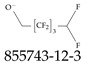	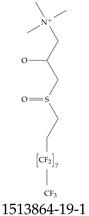	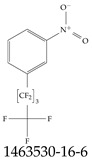
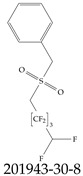	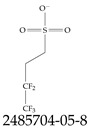	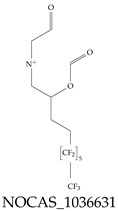	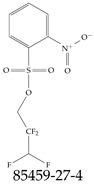
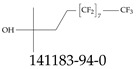	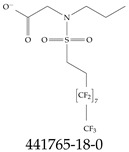	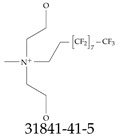	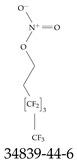
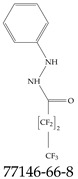	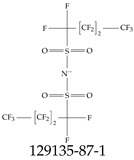	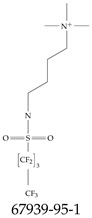	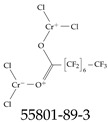

**Table 5:** A selection of the ultrashort-, short-, and long-chain PFAS molecules. Chemical Abstract Service (CAS) numbers are provided for each structure, and the corresponding names are included in Appendix A.

Ultrashort	Short	Long
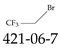	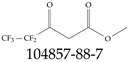	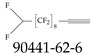
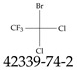	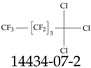	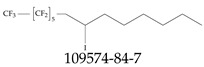
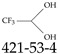	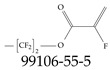	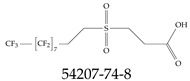
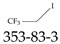	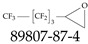	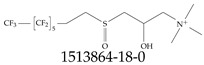
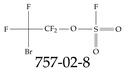	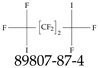	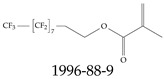

**Table 6:** A summary of some of the PFAS sensors included in this paper. The interested reader could refer to other reports in the literature focused on this topic such as [34,69,70,71].

Sensor	Source
Molecularly Imprinted Polymers	[72,73,74,75,76,77,78,79,80,81,82,83,84]
Quantum Dots	[85,86,87,88]
Nanoparticles	[89,90,91,92]
Large Nanoparticles	[89,90,92]
Biosensors	[93,94,95,96,97]
Metal Organic Frameworks	[98,99]
Covalent Organic Frameworks	[100]

## Data Availability

The raw data supporting the conclusions of this article will be made available upon reasonable request to corresponding author.

## Appendix A. Molecular Structure Names

**Table A1:** A list of PFAS molecules from the EPA PFASStructV5 list, identified with their CAS (Chemical Abstracts Service) number and their corresponding IUPAC names. Note: the chemicals listed with “NOCAS” identifiers refer to structures for which a CAS was either not found or has not been assigned yet, which is sometimes the case with newly detected contaminants or transformation products.

CAS	Name
28462-90-0	2-(Pentafluoroethyl)imidazolidine
2479-75-6	2,3,3,3-Tetrafluoro-2-[1,1,2,3,3,3-hexafluoro-2-(trifluoromethoxy)propoxy]propanoyl fluoride
367-27-1	2,4-difluorophenol
330548-64-6	N-(2,2,3,3,4,4,5,5,6,6,7,7,8,8,9,9,9-Heptadecafluorononanoyl)-L-
124709-43-9	2,3,3,3-Tetrafluoro-2-1,1,2,2,3,3-hexafluoro-3-[(trifluoroethenyl)oxy]propoxypropanenitrile
89516-72-3	6-Azido-2,2,3,3,4,4,5,5-octafluoro-2,3,4,5-tetrahydropyridine
425-19-4	2,2,3,3,4,4,4-Heptafluoro-1-(piperidin-1-yl)butan-1-one
63573-65-9	2-Bromo-2,3-dichloro-1,1,1,4,4,4-hexafluorobutane
20017-53-2	Pentakis(pentafluoroethyl)pyridine
60308-66-9	2,3,3,3-Tetrafluoro-2-(heptafluoropropoxy)propanenitrile
825628-55-5	2,3-Bis[(trifluoromethyl)sulfanyl]propan-1-ol
399-69-9	2,6-Bis(trifluoromethyl)-1H-benzimidazole
66406-80-2	1,1,1,2,2,3,3,4,4-Nonafluoro-4-(prop-1-ene-1-sulfonyl)butane
83865-23-0	(2,2-Dichloro-1,1,2-trifluoroethoxy)(difluoro)acetyl fluoride
184362-57-0	3,3,4,5,5,6,7,8,9-Nonafluoro-2,3,4,5-tetrahydro-1-benzoxepine
14919-09-6	Decafluoroheptanedioic acid
87579-39-3	Methyl 2-[ethoxy(difluoro)methyl]-3,3,3-trifluoropropanoate
NOCAS_1026753	1,1,1,2,2,3,4,5,5,5-Decafluoro-3-propoxy-4-(trifluoromethyl)pentane
88628-84-6	5,5,6,6,7,7,8,8,9,9,10,10,11,11,12,12,12-Heptadecafluorododecanal
89608-30-0	(4,4,5,5,6,6,6-Heptafluoro-2-iodohex-2-en-1-yl)(trimethyl)silane
1140917-25-4	5-(2,2,3,3-tetrafluoro-1-methylcyclobutyl)-1,3,4-thiadiazol-2-amine
1555-33-5	Bis((perfluoroheptyl)methyl) phosphate
74061-31-7	Phenyl(tridecafluorohexyl)iodanium
141206-73-7	1-Isocyano-3,5-bis(trifluoromethyl)benzene
231291-19-3	2-Bromo-3,4,4,4-tetrafluoro-3-(trifluoromethoxy)but-1-ene
855743-12-3	Ammonium 2,2,3,3,4,4,5,5-octafluoropentanolate
1513864-19-1	3-(3,3,4,4,5,5,6,6,7,7,8,8,9,9,10,10,10-Heptadecafluorodecane-1-sulfinyl)-2-hydroxy-N,N,N-trimethylpropan-1-aminium
1463530-16-6	1-Nitro-3-(undecafluoropentyl)benzene
201943-30-8	[(2,2,3,3,4,4,5,5-Octafluoropentane-1-sulfonyl)methyl]benzene
2485704-05-8	2:2 Fluorotelomer sulfonate
NOCAS_1036631	Dimethyl[3-(perfluorohexyl)-2-(formyloxy)propyl](2-oxoethyl)azanium
85459-27-4	2,2,3,3-Tetrafluoropropyl 2-nitrobenzene-1-sulfonate
141183-94-0	5,5,6,6,7,7,8,8,9,9,10,10,11,11,12,12,12-Heptadecafluoro-2-methyldodecan-2-ol
441765-18-0	N-[(Perfluorooctyl)ethylsulfonyl]-N-propylglycine lithium salt
31841-41-5	3,3,4,4,5,5,6,6,7,7,8,8,9,9,10,10,10-Heptadecafluoro-N,N-bis(2-hydroxyethyl)-N-methyldecan-1-aminium iodide
34839-44-6	3,3,4,4,5,5,6,6,6-Nonafluorohexyl nitrate
77146-66-8	2,2,3,3,4,4,4-Heptafluoro-N’-phenylbutanehydrazide
129135-87-1	Potassium bis(1,1,2,2,3,3,4,4,4-nonafluorobutane-1-sulfonyl)azanide
67939-95-1	N,N,N-Trimethyl-3-[(1,1,2,2,3,3,4,4,4-nonafluorobutane-1-sulfonyl)amino]propan-1-aminium iodide
55801-89-3	Chromium chloride-perfluorooctanoic acid complex
421-06-7	2-Bromo-1,1,1-trifluoroethane
104857-88-7	Methyl 4,4,5,5,5-pentafluoro-3-oxopentanoate
90441-62-6	3,3,4,4,5,5,6,6,7,7,8,8,9,9,10,10,11,11-Octadecafluoroundec-1-yne
42339-74-2	1-Bromo-1,1-dichloro-2,2,2-trifluoroethane
14434-07-2	1,1,1-Trichloro-2,2,3,3,4,4,5,5,5-nonafluoropentane
109574-84-7	1,1,1,2,2,3,3,4,4,5,5,6,6-Tridecafluoro-8-iodotetradecane
421-53-4	2,2,2-Trifluoroethane-1,1-diol
99106-55-5	1,1,2,2-Tetrafluoropropyl 2-fluoroprop-2-enoate
54207-74-8	3-[(3,3,4,4,5,5,6,6,7,7,8,8,9,9,10,10,10-Heptadecafluorodecyl)sulfonyl]propanoic acid
353-83-3	1,1,1-Trifluoro-2-iodoethane
89807-87-4	2-(Nonafluorobutyl)oxirane
1513864-18-0	2-Hydroxy-N,N,N-trimethyl-3-(3,3,4,4,5,5,6,6,7,7,8,8,8-tridecafluorooctane-1-sulfinyl)propan-1-aminium
757-02-8	2-Bromo-1,1,2,2-tetrafluoroethyl sulfurofluoridate
375-50-8	1,1,2,2,3,3,4,4-Octafluoro-1,4-diiodobutane
1996-88-9	3,3,4,4,5,5,6,6,7,7,8,8,9,9,10,10,10-Heptadecafluorodecyl 2-methylprop-2-enoate
